# Surgical intervention for vertebral metastases may benefit lung cancer patients no less than other patients: a retrospective study

**DOI:** 10.1186/s13256-016-1157-3

**Published:** 2017-01-04

**Authors:** Takashi Kobayashi, Naohisa Miyakoshi, Toshiki Abe, Eiji Abe, Kazuma Kikuchi, Yoichi Shimada, Seiko Matsumoto, Shin Fukui

**Affiliations:** 1 Department of Orthopedic Surgery, Akita Kousei Medical Center, 1-1-1 Iijima, Nishifukuro, Akita 011-0948 Japan; 2 Department of Orthopedic Surgery, Akita University Graduate School of Medicine, 1-1-1 Hondo, Akita, 010-8543 Japan; 3 Department of Anesthesiology, Akita Kousei Medical Center, 1-1-1 Iijima, Nishifukuro, Akita 011-0948 Japan; 4 Department of Respiratory Medicine, Akita Kousei Medical Center, 1-1-1 Iijima, Nishifukuro, Akita 011-0948 Japan

**Keywords:** Lung cancer, Metastatic vertebral tumor, Revised Tokuhashi score, Prognosis, Survival period

## Abstract

**Background:**

Spinal metastasis is considered to have a worse prognosis in lung cancer than in other cancers, but recent clinical studies report improved overall survival of lung cancer. We compared the postoperative prognoses of vertebral metastatic tumors from lung with other types of cancer.

**Methods:**

From 2011 to 2015, 31 Japanese patients (mean age 73 years, range 55–88 years; 19 males, 12 females) underwent surgery for spinal metastasis at our center. We observed patients retrospectively in March 2016, dividing them into groups by cancer type: lung (LK group, *n* = 10); prostate, breast, or thyroid (PB group, *n* = 12); and other (OT group, *n* = 9). We compared survival and revised Tokuhashi score, which provides a basis for choosing a treatment course. Neurologic status was graded before and after surgery using the Frankel system.

**Results:**

Mean follow-up was 16.5 months (range 1–62 months). Only seven of 31 patients (22.6%) were alive at final follow-up. Frankel grade significantly improved postoperatively only in the LK (*P* = 0.01) and PB (*P* = 0.048) groups. Revised Tokuhashi score differed across groups (*P* < 0.0001), and was significantly lower in the LK group than in the PB group (*P* = 0.00) and OT group (*P* = 0.02). Postoperative survival was significantly shorter in the LK group than in the PB group (*P* = 0.01) but did not differ between the LK and OT groups.

**Conclusions:**

The revised Tokuhashi score may underestimate the survival of lung cancer patients, who may derive the same benefit from surgical intervention as those with vertebral metastasis from other cancer types.

## Background

Lung cancer is the third most common cancer, and the most common cause of cancer death in Japan [1]. However, overall survival of lung cancer has recently improved [2]. Bone metastases from lung cancer are common, affecting 36% of patients with advanced lung cancer [3]. The spine is the most common site of metastatic lesions, which can impair mobility and functional independence [3]. On histology, adenocarcinoma and squamous cell carcinoma are associated with higher and lower risks of developing bone metastases [4]. Surgery is the most effective method of pain relief and recovery from palsy due to paraparesis from vertebral metastasis, and is therefore the treatment of choice when survival longer than 3 months is predicted [5, 6]. The treatment strategy and procedure should be based on life expectancy [7–9]; the revised Tokuhashi score is generally used to evaluate the prognosis of patients with a metastatic vertebral tumor before considering surgical treatment [10–18]. The revised Tokuhashi score is based on six parameters, including patient condition, number of bone metastases outside spine, metastasis to major organs, primary site, and palsy (Table 1). “Palsy” is evaluated using Frankel scores. Frankel scores are defined as follows: A, complete neurological injury; B, preserved sensation only; C, preserved motor, nonfunctional; D, preserved motor, functional; E, normal (no neurological injury). Proper use of the revised Tokuhashi score to determine whether, on the basis of life expectancy, a patient is a surgical candidate can help surgical decision-making in patients with spinal metastases [16–18]. The prognosis of lung cancer metastatic to the spine is considered worse than that of other cancers with spinal metastases [7–9, 19]. However, recent clinical studies have shown improvement in the overall survival of lung cancer [2]. This progress has been facilitated by the introduction of new drugs, and by patient selection based on the recognition that different histological subtypes and driver mutations determine the biology of these malignancies and predict drug efficacy [20]. Therefore, whether the revised Tokuhashi score properly evaluates the prognosis of lung cancer has been called into question. For patients with vertebral metastases, we choose surgery when survival greater than 3 months is expected based on either the original or an unknown malignancy.

**Table 1:** Modified Tokuhashi score

Prognosis parameter	Score
General condition	
Poor (performance status 10–40%)	0
Moderate (performance status 50–70%)	1
Good (performance status 80–100%)	2
Number of bone metastases outside spine	
>2	0
≤2	1
0	2
Metastasis to major organs	
Non-removable	0
Removable	1
None	2
Primary site	
Lung, osteosarcoma, stomach, bladder, esophagus, pancreas	0
Liver, gallbladder, unidentified	1
Other	2
Kidney, uterus	3
Rectum	4
Thyroid, breast, prostate, carcinoid tumor	5
Palsy	
Complete (Frankel A, B)	0
Incomplete (Frankel C, D)	1
None (Frankel E)	2

The purpose of this study was to compare the prognoses of surgically treated patients with a vertebral metastatic tumor from lung versus other cancers, and to determine whether the revised Tokuhashi score properly evaluates the prognosis of lung cancer.

## Methods

Thirty-three consecutive Japanese patients underwent surgery for treatment of spinal metastases at Akita Kousei Medical Center from April 2011 to September 2015. Thirty-one patients (mean age 73.4 years, range 55–88 years; 19 males, 12 females) were included in the study. Two patients were removed because of a lack of clinical data. We observed patients retrospectively in March of 2016. The primary lesions were lung (*n* = 10), and other (*n* = 21). Lung cancers were categorized by histology as non-small-cell lung cancer in nine patients and small-cell lung cancer in one patient. Preoperative assessments included medical history, history of primary tumors, and spinal magnetic resonance imaging. Expected survival >3 months was used as a criterion for choosing surgical treatment [5, 6]. Patient survival and physical and neurological status were noted. The prognosis was evaluated retrospectively using the revised Tokuhashi score. Neurological status was graded before and after surgery using the Frankel system [21]. The postoperative Frankel grade was assigned 1–2 months after surgery, at which time the neurological status was likely to be maximal.

Cases were divided into three groups: lung cancer (LK group, *n* = 10); prostate, breast, or thyroid cancer (PB group, *n* = 12); and other (OT group, *n* = 9), and revised Tokuhashi score and survival periods were compared. Patients’ profiles are shown in Table 2. Survival rates were compared using Kaplan–Meier analysis and the log-rank statistic. All data are expressed as a mean ± standard deviation. All tests of significance were two-tailed, and differences with a *P* value <0.05 were considered statistically significant. All statistical analyses were performed with EZR (Saitama Medical Center, Jichi Medical University, Saitama, Japan), which is a graphical user interface for R (The R Foundation for Statistical Computing, Vienna, Austria).

**Table 2:** Patient profiles

	Age (years)	Sex	Primary tumor type	Revised Tokuhashi score							Complication	Survival period (month)	Preoperative Frankel score^a^	Postoperative Frankel score^a^	Initial stage^b^
				General condition	Extraspinal bone metastases	Vertebral metastases	Internal organ	Primary site	Palsy	Tokuhashi score					
LK															
1	78	f	Lung	0	2	0	2	0	1	5		9	C	D	3
2	81	f	Lung	0	2	1	2	0	1	6		35	C	D	4
3	87	f	Lung	0	2	2	0	0	1	5		4	C	D	1
4	72	m	Lung	0	2	0	2	0	1	5		3	C	D	4
5	60	f	Lung	1	2	1	2	0	1	7		26	D	D	4
6	86	f	Lung	0	2	0	2	0	1	5		12	C	D	4
7	82	m	Lung	0	1	0	0	0	0	1	infection	1	A	A	3
8	72	m	Lung	0	1	2	2	0	1	6		3	C	C	4
9	65	m	Lung	1	2	0	0	0	1	4		8	C	D	4
10	69	f	Lung	0	1	0	2	0	1	4		7	C	D	4
Average	75.2 ± 9.1			0.2 ± 0.4	1.7 ± 0.5	0.6 ± 0.8	1.4 ± 1.0	0	0.9 ± 0.3	4.8 ± 1.6		10.8 ± 11.1			
PB															
11	76	m	Prostate	0	2	2	2	5	1	12		62	C	D	4
12	74	m	Prostate	1	2	1	2	5	1	12		11	D	D	3
13	68	m	Thyroid	1	2	2	2	5	1	13		53	D	D	4
14	84	f	Breast	0	2	1	2	5	1	11		44	C	D	1
15	55	f	Breast	1	0	2	2	5	1	11		40	D	D	4
16	70	m	Prostate	0	1	2	2	5	0	10		24	B	D	4
17	72	m	Prostate	1	2	0	2	5	1	11	infection	27	D	D	4
18	66	f	Thyroid	1	2	1	2	5	1	12		26	D	D	4
19	88	m	Prostate	0	2	0	2	5	0	9		10	A	A	4
20	62	f	Breast	2	2	2	2	5	1	14		26	D	D	4
21	58	m	Prostate	0	2	0	0	5	1	8		3	C	D	4
22	73	m	Prostate	0	1	0	2	5	1	9		5	C	D	4
Average	70.5 ± 9.7			0.6 ± 0.7	1.7 ± 0.7	1.1 ± 0.9	1.8 ± 0.6	5	0.8 ± 0.4	11.0 ± 1.8		27.6 ± 19.0			
OT															
23	71	f	Rectum	0	2	2	0	4	1	9		19	D	D	2
24	76	m	Kidney	0	2	2	2	3	1	10		8	D	D	1
25	71	m	Kidney	0	2	0	0	3	1	6		7	C	D	4
26	83	m	Rhabdomyosarcoma	0	2	2	2	2	1	9		3	C	C	4
27	78	m	Kidney	0	2	0	2	3	1	8	infection	5	C	C	4
28	87	m	Unknown	0	2	2	2	1	1	8		8	C	D	4
29	62	m	Colon	0	2	0	0	2	1	5		2	C	C	4
30	70	m	Colon	0	1	1	0	2	1	5		10	D	D	3
31	79	f	Multiple myeloma	0	2	0	2	2	1	7		9	C	C	4
Average	75.2 ± 7.6			0	1.9 ± 0.3	1.0 ± 1.0	1.1 ± 1.1	2.4 ± 0.9	1	7.4 ± 1.8		7.9 ± 5.0			

*LK* lung cancer group, *PB* prostate, breast, or thyroid cancer group, *OT* other cancers group, *m* male, *f* female

^a^Frankel scores: A, complete neurological injury; B, preserved sensation only; C, preserved motor, nonfunctional; D, preserved motor, functional; E, normal (no neurological injury)

^b^Initial stage: 1, localized; 2, early locally advanced; 3, late locally advanced; 4, metastasized

## Results

Clinical data for all patients, including primary tumor type and location of metastases, are presented in Table 2. All patients had neurological deficits. Mean follow-up was 16.5 months (range 1–62 months). At final follow-up, seven of the 31 patients were alive.

Group comparisons of age, sex, and initial stage were performed with the Kruskal–Wallis test [22], which is a nonparametric method for testing, and there was no significant difference among the groups.

Overall, Frankel classifications improved postoperatively (*P* = 0.00). The mean revised Tokuhashi score was 8.0 (range 1–14). Regarding surgical interventions, total *en bloc* spondylectomy was performed in three patients, anterior spinal decompression and instrumentation in one patient, posterior spinal decompression and instrumentation in 25 patients, and laminectomy alone in two patients.

The Frankel classification improved significantly preoperatively to postoperatively in the LK (*P* = 0.01) and PB (*P* = 0.047) groups, but not in the OT group.

Mean revised Tokuhashi scores for each group are presented in Table 2. Group comparisons of the revised Tokuhashi score were performed with the Kruskal–Wallis test. The revised Tokuhashi score differed significantly across groups (Kruskal–Wallis chi-squared = 22.1, degrees of freedom = 2, *P* < 0.0001). Pairwise comparisons using the Mann–Whitney U test revealed the Tokuhashi score of the LK group to be significantly lower than that of both the PB group (*P* = 0.00) and OT group (*P* = 0.02). There was no significant difference in the survival period with initial stages of “localized” or “early locally advanced” carcinoma compared with “late locally advanced” or “metastasized” carcinoma (Mann–Whitney U test).

**Fig. 1:**
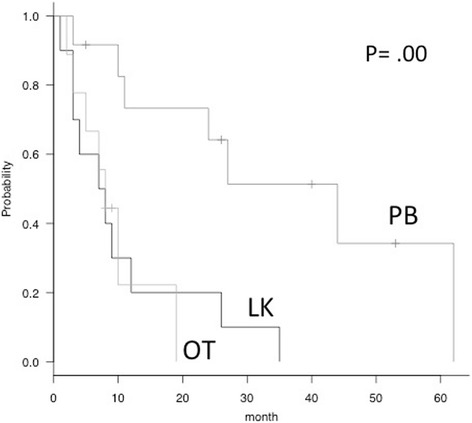
Survival curves of patients with spinal metastases from lung (*LK*) versus prostate, breast, or thyroid primary tumor (*PB*) versus other cancers (*OT*). The postoperative survival period differed significantly among the three groups (*P* = 0.00). Pairwise comparisons using the log rank test revealed the survival period of the LK group to be significantly shorter than that of the PB group (*P* = 0.01), but there was no difference between the LK and OT groups

Mortality after the operation was compared among groups by applying Kaplan–Meier analysis and the log-rank statistic. The postoperative survival period differed significantly among the three groups (*P* = 0.00, Fig. 1). Pairwise comparisons using the log rank test revealed the survival period of the LK group to be significantly shorter than that of the PB group (*P* = 0.01), but there was no difference between the LK and OT groups (Fig. 1). The median survival time after the surgical treatment was 7.5 months in the LK group, 44.0 months in the PB group, and 8.0 months in the OT group.

## Discussion

The prognostic factors associated with spinal metastases are unclear. Surgery can improve mechanical stability, cord compression, and pain, and may be considered when a patient has a life expectancy of >3 months [5, 6]. Palliative surgery for spinal metastasis can improve the quality and length of life [23]. The revised Tokuhashi score is an important and effective tool for considering the prognosis of patients with a metastatic vertebral tumor [10–18]. Spinal metastases are considered to carry a worse prognosis in patients with lung cancer compared with other cancers [7–9, 19]; therefore, conservative treatment is selected for many lung cancer patients. However, improvements in chemotherapy, radiotherapy, and hormonal therapies have led to increased survival times for patients with lung cancer [2, 20].

Our results show that the postoperative survival period after surgical treatment of vertebral metastases was significantly shorter in the LK group than in the PB group, but did not differ between the LK and OT groups, even though the revised Tokuhashi score of the LK group was significantly lower than that of the PB and OT groups.

The present study highlights two important clinical issues. First, some cases of vertebral metastasis from lung cancer can be expected to have a long survival period. Patients with a revised Tokuhashi score of 0–8 are expected to survive <3 months. In the revised Tokuhashi scoring system, lung cancer is assigned 0 points, giving a low score to a lung cancer patient with Frankel C palsy. However, a lack of correlation between the revised Tokuhashi score and the survival period has been reported for patients with spinal metastases of lung cancer [24–26]. Ogihara et al. recommended surgical treatment of spinal metastases from lung cancer for patients without hypercalcemia or hypoalbuminemia [24]. Bilsky et al. showed that, when considering surgical intervention, patient-by-patient assessment may be more important than the result of a scoring instrument such as the Tokuhashi score [27].

Second, the present study demonstrates that, when considering treatment of vertebral metastasis of lung cancer, surgical treatment may be appropriate even if the revised Tokuhashi score is <8 points. Chemotherapy, radiotherapy, and surgery are three treatment options for spinal metastases; of these, surgery is most effective for early mobilization and return to functional ambulation, but it is also the most invasive treatment and risks complications such as infection and worsening of palsy. However, only one of the 11 lung cancer patients in the present study had a severe postoperative complication; that patient (patient #7, Table 2) died from a surgical site infection 1 month after surgery. Despite the fears surrounding the invasiveness of surgery and its associated risks, we had a low rate of surgical complication. Hirabayashi et al. showed that postoperative ambulation was associated with a longer survival time after surgery for spinal metastases in patients with lung cancer [23]. Surgical treatment for a vertebral tumor from lung cancer can improve the quality of life and survival time [28]. Therefore, considering the advances made in lung cancer treatment and the results of the present study, we believe that the revised Tokuhashi score requires further revision, and that a primary cancer site score of 0 for lung cancer may no longer be justified. Because the average score for the primary cancer site in the OT group was 2.4, we believe that a score of 2 for lung cancer may be appropriate.

The limitations of the present study were its relatively small number of patients, the heterogeneity of the OT group, and the questionable validity of comparing the LK and OT groups.

## Conclusions

The revised Tokuhashi score may underestimate the survival of lung cancer patients, who could derive the same benefit from surgical intervention as those with vertebral metastasis from other types of cancer.
